# Perceptions of Australians with diabetes‐related foot disease on requirements for effective secondary prevention

**DOI:** 10.1111/ajr.12989

**Published:** 2023-04-24

**Authors:** Aaron Drovandi, Benjamin Crowley, Chanika Alahakoon, Leonard Seng, Malindu E. Fernando, Diane Ross, Rebecca Evans, Jonathan Golledge

**Affiliations:** ^1^ Queensland Research Centre for Peripheral Vascular Disease, College of Medicine and Dentistry James Cook University Townsville Queensland Australia; ^2^ Faculty of Biological Sciences, School of Biomedical Sciences University of Leeds Leeds UK; ^3^ Ulcer and wound Healing consortium (UHEAL), Australian Institute of Tropical Health and Medicine James Cook University Townsville Queensland Australia; ^4^ Faculty of Health and Medicine, School of Health Sciences University of Newcastle Newcastle New South Wales Australia; ^5^ Department of Vascular and Endovascular Surgery, John Hunter Hospital Hunter New England Local Health District, New South Wales Health Newcastle New South Wales Australia; ^6^ Townsville Aboriginal and Islander Health Services Townsville Queensland Australia; ^7^ Department of Vascular and Endovascular Surgery Townsville University Hospital Townsville Queensland Australia

**Keywords:** diabetes, offloading footwear, patient education, peripheral artery disease, qualitative research, secondary care, telehealth

## Abstract

**Introduction:**

Secondary prevention is essential in reducing recurrence of diabetes‐related foot disease (DFD) but is frequently poorly implemented in clinical practice.

**Objective:**

To explore the perceptions of people with diabetes‐related foot disease (DFD) on their self‐perceived knowledge in managing DFD, facilitators and barriers influencing their DFD care, and ideas and preferences for a secondary prevention program.

**Design:**

Sixteen people with a history of DFD from Queensland and Victoria, Australia, underwent semi‐structured interviews. Interviews were audio‐recorded over telephone and transcribed and analysed following a thematic framework. Participants were asked about their experiences and perceptions relating to DFD and factors influencing the care they receive for DFD relevant to the development of a secondary prevention program for DFD.

**Findings and discussion:**

Participants had high self‐perceived knowledge in managing DFD, especially in implementing healthy lifestyle changes and conducting daily foot checks and foot care, though most received support from family members acting as carers. However, issues with access and adherence to offloading footwear, and a lack of clear education received on footwear and other aspects of DFD care were perceived as major barriers. Improved patient education, provided in a consistent manner by proactive clinicians was perceived as an essential part of secondary prevention. Telehealth was perceived positively through facilitating faster care and considered a good adjunct to standard care. Health and technological literacy were considered potentially major barriers to the effectiveness of remote care.

**Conclusion:**

People with DFD require improved access to offloading footwear and education about secondary prevention, which could be provided by telehealth with adequate support.

**What is already known on this subject**

- Secondary prevention is essential in reducing recurrence of diabetes‐related foot disease (DFD) but is frequently poorly implemented in clinical practice.
- The lack of DFD secondary prevention programs means missed opportunities to prevent hospital admissions and amputations.
- This study aimed at identifying the experiences and perceptions of patients with DFD on the elements required in a DFD secondary prevention program.

**What this paper adds**

- Participants had high self‐perceived knowledge in managing their DFD, but most acknowledged they required assistance from family members.
- Participants had major barriers accessing and using offloading footwear, partially caused by lack of clear education on appropriate footwear use.
- Participants indicated the need for psychological support which could by incorporated in a telehealth education and support program.

## 1 INTRODUCTION

Diabetes‐related foot disease (DFD) encompasses a cluster of related foot problems, including foot ulcers, infection and gangrene.^1^, ^2^ Globally, DFD is a leading cause of disability, requirement for medical treatment and reduced health‐related quality of life.^3^, ^4^ Models of DFD care usually focus on treating active foot complications,^2^ though due to the high recurrence rate, the majority of patients need repeated treatment, often leading to multiple repeat hospital admissions.^1^ This can lead to a chronic state of poor health and places a large burden on patients, their caregivers and health systems.^1^ The chronic and recurring nature of DFD promotes depression, disability and reduced life expectancy.^3^, ^5^

Control of modifiable risk factors by offloading high foot pressures, regular monitoring of the foot and long‐term control of blood sugar and dyslipidaemia can reduce the risk of DFD recurrence.^6^ Current guidelines list an overwhelming number of secondary prevention recommendations for DFD, including changes in diet and other lifestyle factors, wearing offloading footwear, daily foot checks and a range of medications and regular appointments with different health professionals.^6^, ^7^, ^8^, ^9^ These may not be practical for all patients, such as for those with physical disability and those experiencing socio‐economic inequality.^2^, ^10^

Currently, secondary DFD prevention is provided by a range of specialists, often through an uncoordinated array of appointments. To the authors' knowledge, there are no well‐established holistic secondary prevention programs for DFD, representing a missed opportunity to reduce DFD recurrence and healthcare burden. The development of such programs require patient input, as the end‐users of the program, to realise their needs and preferences.^11^ As poor access to health services may affect many people with DFD, telehealth may facilitate efficient delivery of such programs,^12^ but how acceptable this is to patients is currently unclear.^13^, ^14^

This study aimed at understanding the needs and preferences of people with DFD for a secondary prevention program. This included how their foot condition was managed, what was missing from their current ideal health care and their capacity to use telehealth for foot care. This study formed part of a larger study, which employed an exploratory, sequential mixed methods design to gather the perceptions of patients and clinicians to co‐design a telehealth secondary prevention program for DFD.^15^

## 2 MATERIALS AND METHODS

### 2.1 Study design

This study used semistructured telephone interviews to gather experiences of Australians with DFD, including their perceptions and preferences for a secondary prevention program. The interviews were conducted between 19th August 2020 and 20th January 2021. Ethics approval for this study was granted by the Townsville Hospital and Health Service Human Research Ethics Committee (HREC/QTHS/53880). The study was reported according to the COnsolidated criteria for REporting Qualitative research (COREQ) checklist.^16^

### 2.2 Recruitment and sampling

Australian adults with a history of DFD were eligible to participate. Participants were encouraged to invite their carer to contribute to the interview discussions due to their valuable experiences of DFD care.^17^ Targeted and purposive snowball sampling techniques were used to identify potential participants. Eligible participants were identified through searching a database maintained by the Queensland Research Centre for Peripheral Vascular Disease at James Cook University, Townsville, Australia, who consented to being contacted about new research studies. Eligible patients were mailed a study information sheet and subsequently contacted by phone to establish interest in participating. Diabetes Australia also distributed information about the study to their members on 11 September 2020. Finally, The Townsville University Hospital and James Cook University distributed the study link through their web and social media pages.

### 2.3 Sample size

The sample size was guided by the concept of thematic saturation, being the point where no new information or themes are observed with additional participants.^18^ Given the narrow focus of the study and previous qualitative research on DFD,^19^, ^20^, ^21^, ^22^ we anticipated that 10 to 20 participants would be sufficient to achieve saturation, at which point data collection would cease. The determination of saturation was achieved through concurrently conducting interviews and analysing the data from the interviews between multiple authors.

### 2.4 Phone interviews

Semistructured telephone interviews were used to ensure participants from geographically disperse areas could participate and to facilitate study completion during the COVID‐19 pandemic. An interview guide was developed (see Appendix A) based on previously published research,^23^, ^24^ a prior survey of Australian patients with DFD, and in collaboration with a sample of patients, health professionals and Aboriginal and Torres Strait Islander representatives. The interview guide aligned with all six elements of the Health Belief Model (HBM)^25^ in order to identify gaps and opportunities in the design of a DFD secondary prevention telehealth program. The HBM describes how perceptions relating to disease influence health‐related behaviours, including perceived (1) susceptibility, (2) severity, (3) benefits, (4) barriers, (5) cues to action and (6) self‐efficacy. For example, where perceived susceptibility to DFD or perceived severity of DFD is low, or where perceived barriers to preventing or managing DFD is high, a secondary prevention program would need to be designed to address these issues. The interview guide was structured into three broad sections: (i) management priorities for DFD; (ii) facilitators and barriers to secondary prevention; and (iii) ideas and preferences for a secondary prevention program.

All participants gave verbal informed consent before participating in the interview. Interviews were conducted by one male researcher (Author 2 [Clinical Research Worker; MPH‐MBA]) who was not involved in treating DFD patients and received training from Author 1 (PhD) who has significant experience conducting qualitative interviews. Two pilot phone interviews with eligible participants were first conducted by Author 2 and reviewed by Author 1 who then instructed on required changes in questioning technique and the use of prompts. Findings from the pilot interviews were included in this study as few changes were made to the interview guide. Interviews were conducted at a time of the participant's choosing, and ranged between 25 and 90 min and were audio‐recorded. There were no repeat interviews conducted, and transcripts were not returned to participants for member checking. Field notes were made during the interview and used in data analysis where needed. No participant had any pre‐existing relationship with the interviewer.

### 2.5 Data analysis

Interview recordings were transcribed verbatim and thematically analysed as advocated by Braun and Clarke (2006), in NVivo (QSR International Pty Ltd.).^26^ Three researchers (Authors 1–3 [MBBS, MPhil]) familiarised themselves with the transcripts, and independently coded using a line‐by‐line open coding process with themes identified using a constant comparison process, as advocated by Corbin and Strauss.^27^ Independently generated themes were reviewed to confirm points of data convergence and reach consensus for data divergence. Illustrative quotes are reported verbatim to support the discussion and identified using a participant number. The 2019 Modified Monash Model was used to classify a participant's remoteness.^28^ This model was developed by the Australian Department of Health and uses population size and geographical remoteness from major cities.

## 3 RESULTS

### 3.1 Sample characteristics

Sixteen participants were interviewed, at which point data saturation was reached, and no additional interviews were conducted. Table 1 details the individual participant characteristics, which included nine men and seven women, aged 48–78, two of whom identified as Aboriginal and most of whom lived in regional areas of Queensland.

**TABLE 1:** Characteristics of the 16 interviewed participants in this study.

No.	Gender; age	Aboriginal and/or Torres Strait Islander	State; rurality	Brief DFD history
1	M; 77	No	QLD; MM2	Sudden ulcer led to major amputation 5 years ago and confinement to wheelchair.
2	M; 77	No	QLD; MM2	Recent ulcers and gangrene leading to minor amputation on one foot; healing ongoing.
3	M; 64	No	QLD; MM2	Improper footwear causing recent ulcers and infection and minor amputation.
4	F; 54	No	VIC; MM1	30‐year DFD history including multiple ulcers, leading to a below‐knee amputation.
5	M; 64	No	QLD; MM5	Multiple lesions turning into ulcers leading to two minor amputations (toes removed).
6	M; 75	No	QLD; MM5	Broken footwear led to ulcer formation and single minor amputation (toe removed).
7	M; 49	No	QLD; MM2	Cellulitis and other infection has led to amputation of three toes; healing ongoing.
8	M; 74	Aboriginal	QLD; MM4	Neuropathy and abrasions due to improper footwear has led to four toe amputations.
9	M; 70	No	QLD; MM2	Recent foot injury led to an infected ulcer; surgically debrided but no amputation.
10	F; 78	No	QLD; MM2	History of painful neuropathy and fungal infections; healing ongoing.
11	F; 76	No	QLD; MM2	Has had a single foot ulcer due to an abrasion which has recently healed.
12	F; 68	No	QLD; MM2	Concurrent infections leading to a single toe amputation.
13	F; 62	No	QLD; MM2	Previous Charcot's foot, and improper footwear causing osteomyelitis; healing ongoing.
14	F; 74	No	QLD; MM2	30‐year DFD history with recurring ankle ulcers; no amputations and healing ongoing.
15	F; 48	Aboriginal	QLD; MM2	Puncture wound leading to infection and toe amputation.
16	M; 75	No	QLD; MM4	Recurring foot ulcer due to improper footwear; no amputations and healing ongoing.

*Note*: ‘Recent’ refers to issues commencing within the 12 months prior the interview.

Abbreviations: F, Female; M, Male; MM, Modified Monash Model category (1—metropolitan centre, 2—regional centre, 4—medium rural town, 5—small rural town); QLD, Queensland; VIC, Victoria.

### 3.2 Key themes generated

**FIGURE 1:**
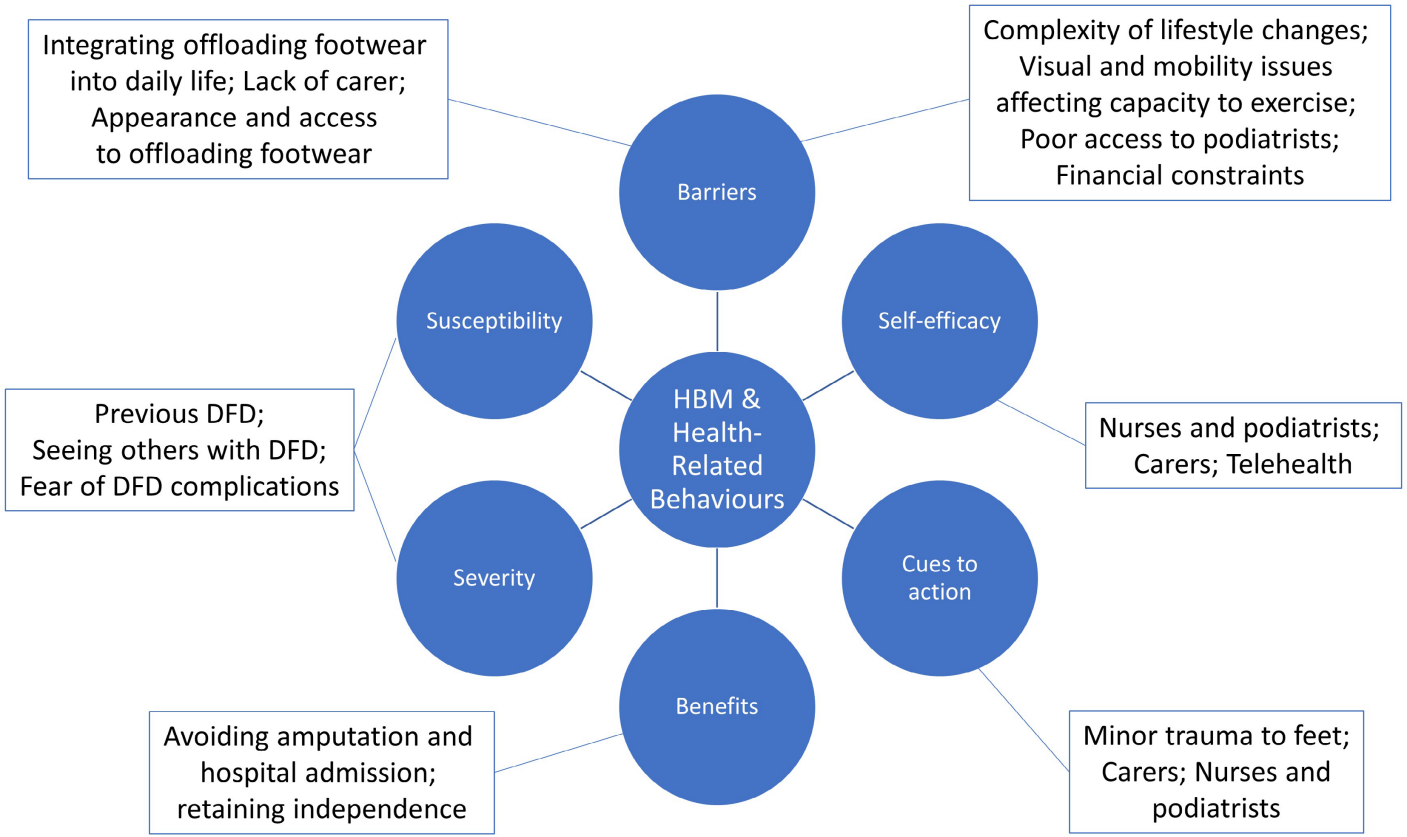
Aligning of subthemes identified with the six components of the Health Belief Model (HBM).

Across the 16 interviews, four overarching themes were identified: (1) management of DFD at home, (2) facilitators and barriers in managing DFD, (3) ideas and preferences for secondary prevention and (4) perceptions of remote care (telehealth) for DFD. Subthemes are identified below and were aligned with the HBM to gain an understanding of recurring issues and how participant perceptions contributed to health‐related behaviours (Figure 1).

#### 3.2.1 Management of DFD at home

**FIGURE 2:**
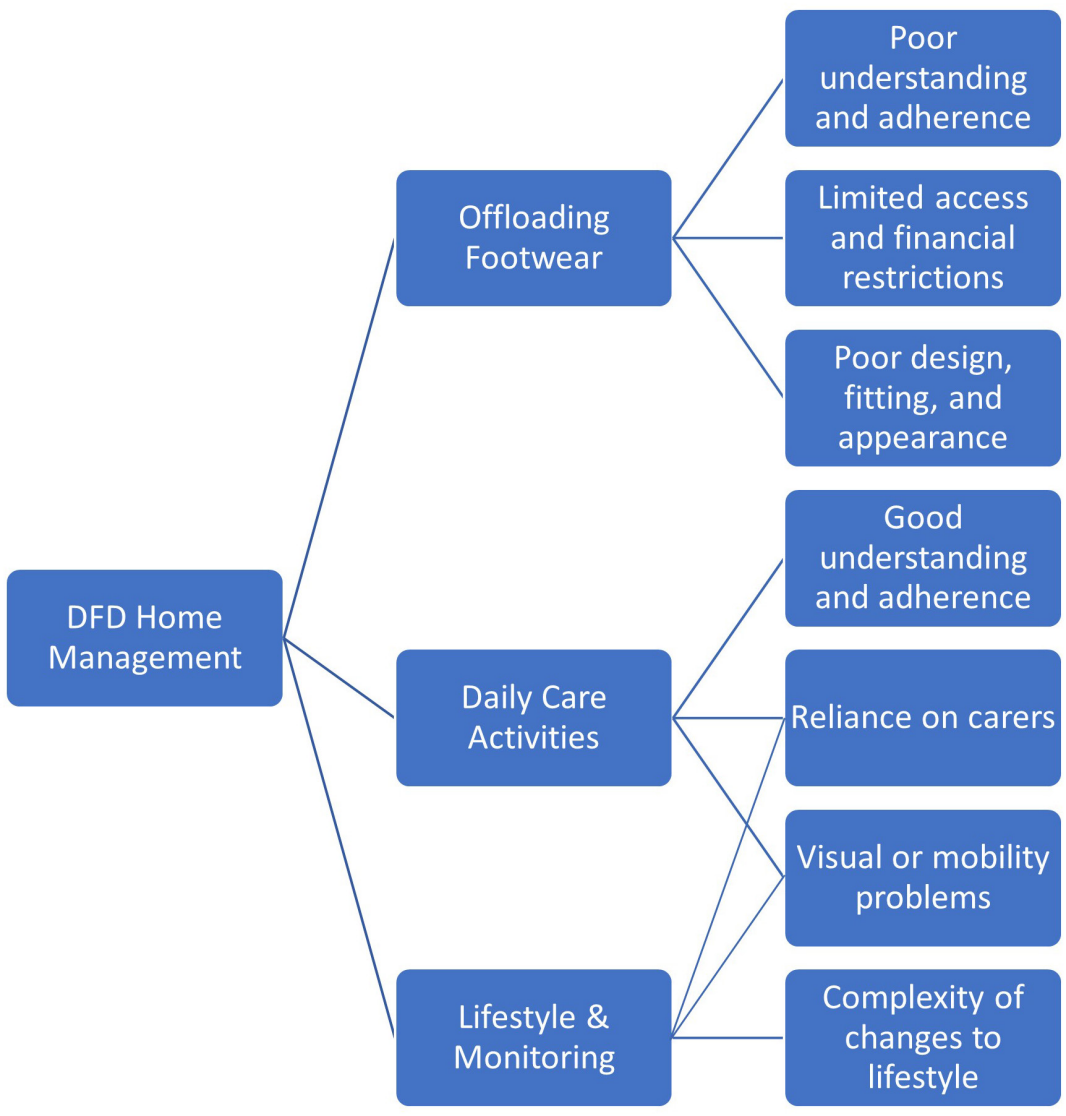
Key themes identified relating to factors impacting on participant foot care in the home environment.

Most participants were aware of the range of activities that should be performed in the home environment to care for their feet and prevent DFD. Several of these activities were perceived as being both important to perform, but difficult to manage. The key management activities included: (1) daily use of offloading footwear, (2) daily foot care and foot checks and (3) lifestyle changes and monitoring (see Figure 2).

##### Offloading footwear

Participants found it challenging to integrate offloading footwear into daily life and wearing footwear whenever upright, as many previously wore inappropriate nonenclosed footwear, or walked barefoot a lot of the time.

They tell me that I should wear shoes all the time. Because I grew up in North Queensland I never wore shoes. And all of a sudden I have to wear shoes all the time and it's a bit boring. (#11)

However, after experiencing DFD, the fear and recognition of the severity of subsequent DFD enforced their motivation to wear prescribed footwear.

Straight out of bed and into, get changed and get into me shoes…[previously] I'm not going to wear shoes unless I have to, but now I don't have an option. (#2)

Despite this recognition of recurrence and knowledge of the protective benefits of offloading footwear, many participants demonstrated suboptimal adherence to appropriate footwear, including within the home environment.

I want to work towards sort of not having to go through that again. And so I am being careful. Every now and again I take a risk and I don't wear my shoes outside… so far nothing bad has happened. (#12)

There were also several complaints relating to offloading footwear. First about the education of patients on what ‘offloading footwear’ means and how to access it.

Protected footwear, it's a horrendous term. It's difficult to know what orthotists mean by that. The ones you can find are very expensive…Tells you what it's going to do, but it doesn't actually tell you where to go to buy them, what exactly they need to be. (#4)

The unsightly appearance of footwear was also raised. This did not appear to lead to poor adherence to wearing the footwear, but did reduce participants' satisfaction.

They really make you stand out in the crowd and you know, I'm not old and I like to think I'm well dressed and professional. And all of that is difficult when you're just wearing runners on the bottom of your feet. (#4)

For some participants, even though they had received offloading footwear, they were not satisfied with the design and fitting, with some stating they believed the footwear made their condition worse, or increased their risk of an injury.

It's just difficult to wear them because they are a slightly different height to the other you know, the other shoe. (#5)

##### Daily foot care and foot checks

Most participants demonstrated a high level of self‐perceived understanding and adherence to a daily foot health routine. Daily and incidental foot checks and regular moisturising were the main activities described.

Well, I do check my foot daily. As I said, I'm vision impaired. You know, I've got a magnified mirror. I have good lighting in my bathroom that sort of thing, (#4)

I find if I stub my foot I'm very worried then and I keep an eye on it and so far nothing bad has happened. (#12)

However, many participants relied on family members acting as carers to perform these duties at least some of the time. This was usually due to having mobility or visual problems which prevented their ability to check their own feet.

It's difficult for me to because of my bad back. It's difficult for me to get down and cut my toenails…my husband cuts them for me…If I suspect that there's anything wrong I get him to inspect my feet closely. (#10)

The absence of this support network at home for some participants made these daily activities more difficult, and led to increased uncertainty and stress about unchecked foot problems.

I can see my toes at 90 degrees, but I can't actually see the whole entire wound on my own. I can check the top half, the front half and most of the bottom it's just parts that I can't see. So if there's something happening there…. (#7)

##### Lifestyle changes and monitoring

Many participants described the importance of lifestyle changes after their diagnosis with diabetes, and more so after their first DFD‐related complication. They also described uptake of home blood‐glucose monitoring to ensure their improved diet was translating to better blood glucose readings.

I've got my blood sugar testing kit. I just get myself into the groove of having to do it. Just doing it because it is necessary to keep it sorted. (#3)

Participants were aware these changes were made in order for them to continue or return to walking normally and avoid hospital admissions and possible amputation.

I don't want any more limbs taken from me. I don't want to be in a wheelchair… And not having somebody care for me you know. I'd like to do things on me own. (#8)

However, the complexity of some of the lifestyle changes such as dietary changes, and difficulties achieving others such as quitting smoking, affected how well participants could integrate these changes successfully into day‐to‐day life. Some lifestyle changes were also not possible to implement, with many describing recommended physical activity as difficult or impossible to achieve due to poor physical health.

I'm not really probably that good with my diet because I've reverted to eating to what I used to eat…Oh and I also smoke I have been a lifetime smoker and it's very difficult habit to break'… probably one of the hardest things is not walking, (#7)

So you get someone that hasn't walked in many years, because I went blind. So I gave up. Absolutely gave up [on exercise]. (#15)

#### 3.2.2 Facilitators and barriers in managing DFD

**FIGURE 3:**
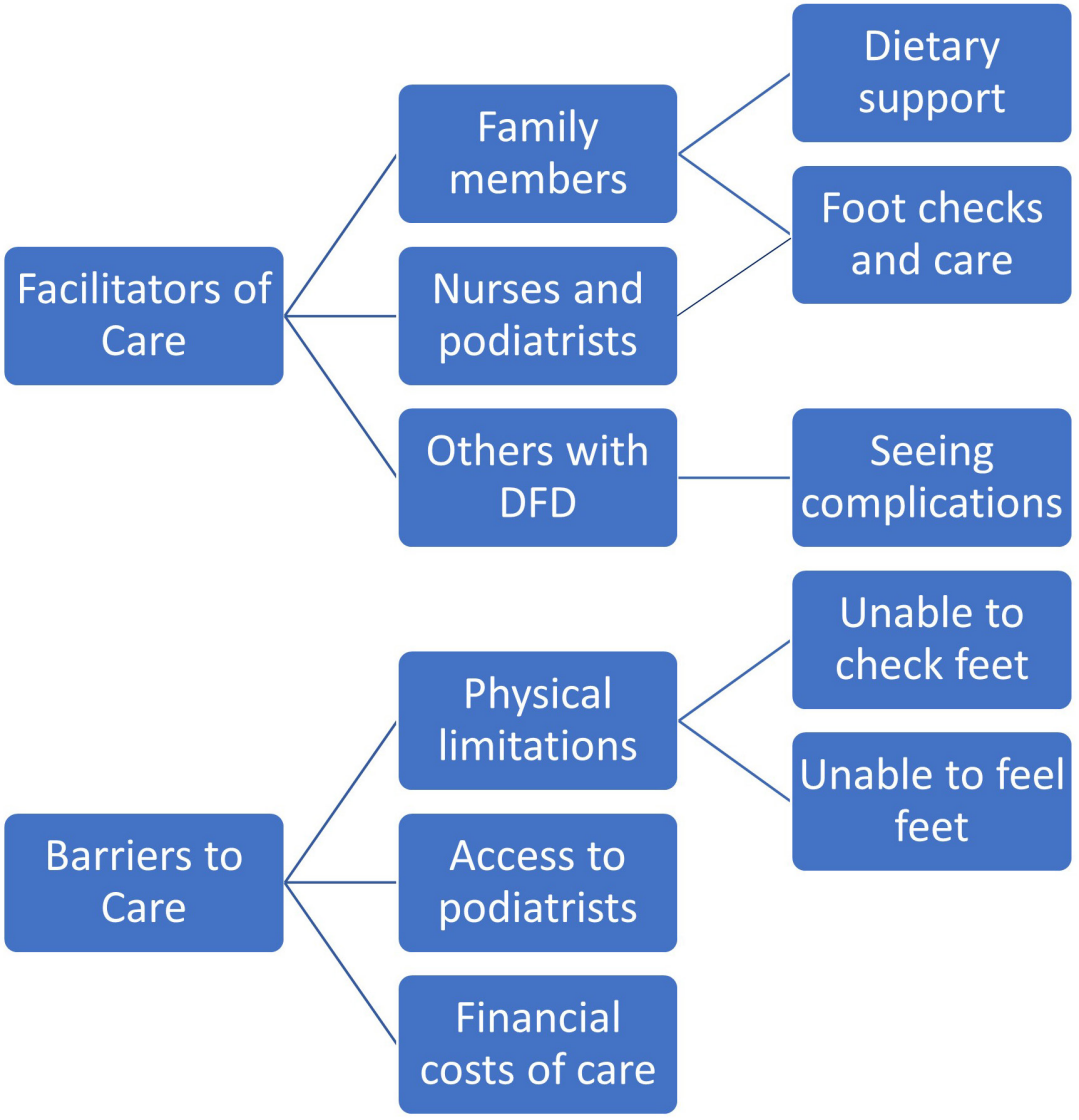
Key themes identified relating to the facilitators and barriers of DFD management.

There were a wide range of factors identified that either facilitated DFD care or were barriers to care (see Figure 3).

##### Facilitators

Support from family members and existing community services were considered the greatest facilitators in managing DFD. Many participants had a spouse act as a carer, without whom they believed they would be far less confident in their DFD management and more likely to experience complications.

Mainly my wife, she watches what I eat. She cooks stuff, a diet that hasn't got a lot of sugar…I visit the podiatrist regularly…the one I go to is pretty good because he happens to be my son. (#1)

So if you are single, it will be a lot harder because it's not just looking after your feet. It's looking after yourself. Like your exercise, your mental health. (#7)

Community nurses and podiatrists made available through access schemes were also seen as invaluable for managing DFD and preventing complications, even for participants who had family members acting as carers.

I'm going to [the high‐risk foot service] three days a week and every second week I'm going out to the big hospital. I find that they're doing a wonderful job looking after me with that. (#2)

Fear of complications of DFD was also raised as a facilitator for improving their own foot care, as it increased many participants' proactivity and motivation to care for their feet.

I haven't got much more [of my foot] to lose there anymore. If it happens again, it could come off at the ankle or below the knee. So that put the jitters into me. So, now everything's going really well at the moment. (#6)

##### Barriers

Participants who lived or were treated in smaller regional towns indicated that there was poor availability of podiatrists.

One of the other bad things I found about the rehab center I went to was you've got a whole ward of people who have had an amputation, mainly due to diabetes, and there was no podiatrist provided to that rehab center. (#4)

Personal issues perceived as major barriers included several forms of physical limitation that affected self‐care, such as neuropathy, immobility and poor vision. These limitations were noted as preventing effective foot self‐care, such as seeing and feeling problems with the feet. These limitations led to their dependence on others, such as family members, for foot care.

Yeah, my vision has been a barrier is making it more difficult, as I say, the lack of feeling in my well now only foot and previously both feet makes it difficult. (#4)

I don't do much for myself because I can't do much. Yeah, I can't look after my feet. I have the nurses come in and do that. (#14)

Financial instability and the cost of therapies for DFD were perceived as a barrier for some participants. Some participants also mentioned they were aware of their relative financial stability compared to others with DFD and the likely struggle of people affording treatment.

I've been lucky that financially it hasn't been a burden. I know the financial cost of a private podiatrist could be a barrier to a lot of people…I can say that seeing a podiatrist was not going to be something on their list of things that were important after they left rehab. (#4)

#### 3.2.3 Ideas and preferences for secondary prevention

**FIGURE 4:**
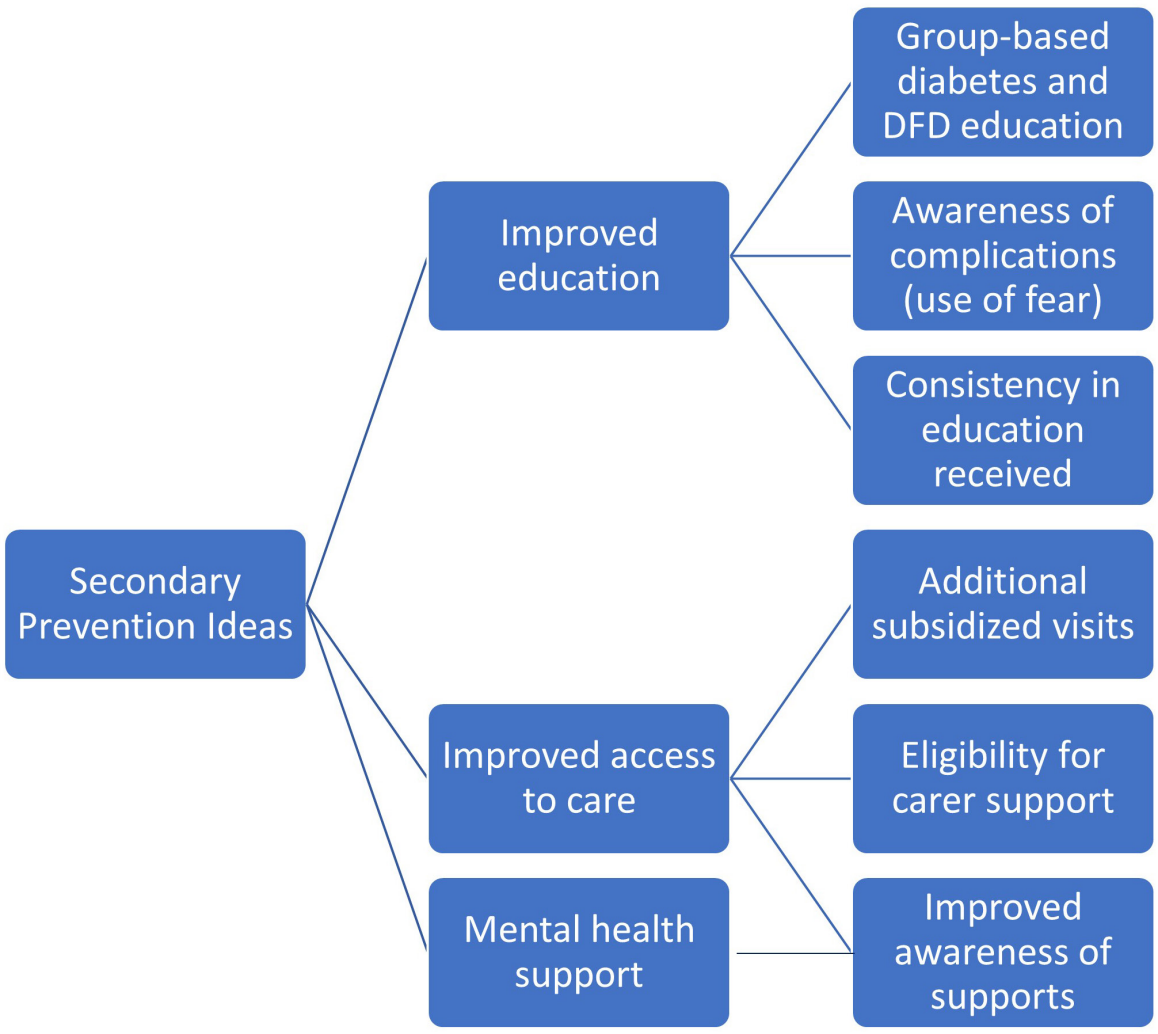
Key themes identified relating to the development of a secondary prevention program for DFD.

Participants were quite vocal in their opinions for secondary prevention, with most recommendations relating to issues that they themselves had experienced when managing their own DFD. Improved patient education, access to care and access to mental health support were the key subthemes raised (see Figure 4).

##### Education

Ensuring adequate patient education on diabetes and related foot complications was at the forefront of interviewees' ideas and preferences.

So I think it's just getting the message across to them how dangerous it is. Once they do get infected…sort of get through to them before it is too late, (#6)

Well, they've got to be educated…when I was diagnosed with diabetes, I had the prior knowledge of having had a grandmother and a mother with diabetes. And so therefore, I was always aware of it. (#10)

Instilling fear through education was believed to be an effective tool in informing people with diabetes on the potential foot‐related complications of their disease.

I'm sure there are people that don't understand that foot problems can be a really, you know, possible factor in diabetes. There is a fine line between putting fear into people and trying to educate them… I'm not sure how much people take that [DFD complications] seriously until something happens. (#4)

Group discussions and patient‐centred workshops were considered an effective medium for the education of people with newly diagnosed diabetes and those at high risk of DFD. This was in part through ‘idea‐sharing’ and part through inducing fear of DFD complications.

The more you talk about things, the more ideas you get. And there are times when you go, “gee I never even thought of that”. (#2)

They need to have…some sort of chat group, either via the Internet or in a community environment where people with like type problems can talk and swap stories. (#3)

Fear was also perceived as being tied to improved motivation in managing DFD.

But there's a big gap between knowing you have to do it and making a habit of it. But if they could be shown that it's a quick thing and I don't know, maybe there does need to be a bit of fear. (#4)

Consistency of education and clear information were also raised as an issue experienced by participants. Receiving a large volume of complex information, coupled with inconsistencies between clinicians, was described by many participants as a barrier to their ability to care for their DFD at home.

All the things are described in quite an abstract manner. Make sure you perform a foot check every day. What do you mean by that? How do you do that if you're an obese person or like me vision impaired? How do you do that when there are a lot of people cannot reach their feet? (#4)

Having proactive clinicians with good bedside manner that related recommendations to the individual patient was also raised by participants. Several participants found their experience could be improved with enhanced clinician communication skills.

I think GPs or podiatrists need to be more hands on in how things should happen. You know, and even like everyday [activities]…because everybody says yes to everything they're told to do. But not everyone can fit everything into their lives. (#4)

##### Improving access to care

Access to existing care services was perceived as a shortcoming of the current system. This included the limited number of government‐subsidised visits allowed per year per person and eligibility for a part‐time funded carer.

So the five [subsidised] visits a year I think are probably not enough for a diabetic person…I think once a month so twelve visits a year would probably be better. (#12)

I've got my wife caring for me…which is fantastic. But people maybe a bit older, in the same situation they wouldn't be able to cope if they didn't have a partner or some access to some home services. (#7)

Improving the awareness of patients about these services and the affordability of these services was also perceived as needed.

I did not know there was such a thing as the high‐risk foot clinic. Now, perhaps I hadn't looked hard enough. Perhaps my podiatrist should have told me these things existed. (#4)

##### Mental health support

When asked about what could assist in the management of DFD at home, many participants described suffering from adverse mental health issues.

It's broken my heart that I cannot get out and do anything because like yesterday I thought I'd get out and I do a bit of gardening…but I had a fall in the front yard. (#2)

Although some participants had received appropriate support, mental health issues were seen as being largely unmet, including the lack of psychological support services for people with DFD, especially those who had undergone amputations.

The lack of psychological support, I spent a couple of months in a public rehabilitation center…But absolutely appalling in helping people deal with the loss of a limb… I was really battling with the loss of a limb. I was made to feel like I should be a bit more resilient. (#4)

#### 3.2.4 Perceptions of remote care for DFD

The final interview topic largely found positive perceptions of telehealth as a method for supporting people with DFD. Participants believed that telehealth would facilitate faster care and keep people ‘on track’ with their daily self‐care and monitoring.

If a person does have a weeping cut or something, rather than not doing anything, if somebody used a camera and said look, you need to keep a dressing on that and see a doctor as soon as possible. (#12)

The way it is now I've got to wait and wait until my doctor's got a chance to see me. (#15)

They also thought it would be a good medium for providing patient education and conducting peer support groups.

You can ask any questions in a comfortable environment where a lot of people are less intimidated, even [compared to] just being in the waiting room you know. (#7)

There were, however, also concerns raised about remote health care, with several participants believing that DFD management requires face to face interactions between patients and clinicians, and that patients would struggle to operate the required technologies.

I believe that hands on is always the way to go. If you can't see what you're looking at, you don't know what you're doing. (#2)

The majority of people my age, are not computer literate…And a lot of the people that are retired, retirees they either haven't got a computer or they can't afford to have a computer or they're not interested in computers. (#10)

## 4 DISCUSSION

This study of Australian DFD patients used the HBM to explore patient issues related to managing DFD, identified barriers and facilitators to care, and highlighted multiple areas of care for which participants required more assistance. Many participants spoke about their fear of DFD recurrence and the consequence of amputation.^29^ The burden and challenges of performing daily foot checks and obtaining and wearing appropriate footwear were also indicated. Finally, participants raised the need for improved access to podiatrists, help with foot monitoring, more education and psychological support. There have been few previous studies examining the lived experiences of people with DFD.^19^, ^20^, ^21^, ^22^ In the current study, participants raised several issues that could be improved in current practice including patient education, care access and mental health support, as well as recommendations to implement these improvements.

Through aligning the subthemes identified with the HBM, it was apparent that participants were acutely aware of their personal risk of DFD complications and the severity of these complications. It is likely this is due to the nature of DFD and how it clearly impacts on health and functioning, as opposed to more subtle chronic diseases. However, this awareness may not translate to effective self‐care behaviours, as identified by participants in this study and in previous research.^30^ Studies investigating behavioural change strategies for people with DFD, such as motivational interviewing,^31^ have reported inconsistent efficacy, with further research needed to clarify the most effective means to support patients' self‐care.^32^ No participants in this study indicated they were satisfied with the education they had received about DFD, with the sources of education and consistency between sources raised by participants as ongoing issues. Several studies have evaluated the effectiveness of group‐based education for DFD in reducing foot‐related complications, including the use of photographs of diseased feet and group discussions.^33^, ^34^, ^35^, ^36^ These studies have had mixed results, with hard endpoints such as ulcer incidence showing no change, and no clearly effective method for individual or group‐based education identified.^33^ Group‐based education sessions were raised as a potential avenue for reinforcing the need for preventive foot care by illustrating the risk of complications. These group‐based sessions were also suggested by participants to facilitate the development of support networks and idea‐sharing on effective foot care. Similar to a previous study,^22^ DFD participants requested that health professionals make efforts to understand the difficulties experienced by people with DFD and tailor their communication to reflect these experiences and patient needs.

A range of barriers were also identified, which ideally would be mitigated in a secondary prevention program to ensure people with DFD have adequate support and access to care. Personal, physical and financial limitations were at the forefront of participants' concerns. Previous research indicates that these concerns are common for people with DFD,^19^, ^20^ with limited resources also affecting capacity of healthcare systems to provide services.^15^, ^21^ Improving access to care, such as podiatrist availability, alongside financial subsidies is a critical component of a DFD secondary prevention program, through directly influencing uptake and adherence to the program.^15^ Mental health support was noted to be absent from current care, with participants particularly requiring psychologically support in processing the trauma associated with a lower‐limb amputation.^22^ Currently, in Australia, there is a substantial deficit in funding provided for preventative care of people with DFD. For example, podiatry visits are capped at a limited number per year, publicly funded footwear is limited to a select group and often there are shortages in supply and delays in receiving these. There is a lack of streamlined triage and appropriate early intervention for people presenting with DFD to regional, remote and rural centres.^37^

Technology for health care may assist in overcoming some of these issues, as it can increase patient self‐efficacy to perform daily foot care management, and act as an avenue for group‐based educational activities.^14^ For example, in a recent meta‐analysis, hypertension, dyslipidaemia and hyperglycaemia were more effectively managed with remote management compared with conventional care.^38^ Telehealth as a medium for health care has expanded considerably in recent years.^13^ It can support DFD patients by reducing travel time and time off work associated with multiple visits to health professionals, provide faster access to podiatrist advice and care, serve as a medium for mental health support, and point of care for acute foot‐related issues.^14^ While telehealth may serve as a support system for risk factor management and some specific aspects of DFD care, it is not a panacea, particularly as previous research has raised the limitations of telehealth for DFD care. Limitations might include the need for physical presence with a podiatrist for certain healthcare activities, and the poor capacity for some people to use technologies required for telehealth, particularly the elderly.^14^ Hence, whether remote management approaches are effective in individuals at the highest risk of complications, such as amputation and cardiovascular events, remains to be tested in large trials.^38^

### 4.1 Strengths and limitations

The interview guide was rigorously developed with input from previous published research, several health professions and patients with DFD including Aboriginal and Torres Strait Islander representatives. The interview guide was also developed using the HBM to ensure the results aligned with validated principles on patient perceptions and health behaviours. Also, multiple trained staff were involved in the thematic analysis to ensure the themes and subthemes derived were reflective of participant perceptions. However, several limitations should also be acknowledged. People who participate in research are generally more motivated and engaged in their disease management, making their perceptions likely to be different to those who do not engage in research. Also, most participants had family members acting as carers, increasing their self‐perceived capacity to manage their DFD. Finally, the perceptions of Australians with DFD may not be reflective of the lived experiences of people with DFD outside of Australia.

## 5 CONCLUSIONS

DFD places an enormous burden on patients, their careers and healthcare systems, with advancements in secondary prevention required to reduce these burdens. This study generated themes describing the strengths and shortcomings of current management strategies for people with DFD. Future management of DFD care requires improvements in education for patients and carers, and addition of new supports such as mental health care. A holistic, collaborative and multilevel approach is required involving patients, researchers, clinicians, government organisations and nongovernment organisations in improving outcomes for people with DFD in Australia.

## AUTHOR CONTRIBUTIONS

**Aaron Drovandi:** Conceptualization; methodology; data curation; formal analysis; writing – original draft; writing – review and editing; investigation. **Benjamin Crowley:** Methodology; investigation; writing – review and editing; data curation. **Chanika Alahakoon:** Methodology; data curation; writing – review and editing; investigation. **Leonard Seng:** Methodology; investigation; data curation; writing – review and editing. **Malindu Fernando E:** Investigation; writing – review and editing; methodology; data curation. **Diane Ross:** Methodology; conceptualization; investigation; writing – review and editing. **Rebecca Evans:** Conceptualization; methodology; writing – review and editing; validation. **Jonathan Golledge:** Conceptualization; methodology; investigation; funding acquisition; writing – original draft; writing – review and editing; project administration; resources; supervision.

## FUNDING INFORMATION

This work was supported by the Townsville Hospital and Health Service Study, Education and Research Trust Account (SERTA) Fund, a Tropical Australian Academic Health Centre Seed grant and the Queensland Government. JG holds a senior clinical research fellowship from the Queensland Government, Australia, and funding from the Australian National Health and Medical Research Council, Heart Foundation and Medical Research Futures Fund. The funders played no role in study design, conduct, data collection, analysis and interpretation, and did not assist in preparation or review of this manuscript.

## CONFLICT OF INTEREST STATEMENT

The authors declare that there are no conflicts of interest.

## ETHICAL APPROVAL

This study was conducted according to the Declaration of Helsinki and informed consent was received by all participants. Ethics approval for this study was granted by the Townsville Hospital and Health Service Human Research Ethics Committee (HREC/QTHS/53880).

## Data Availability

Data are available from the corresponding author upon reasonable request.

## APPENDIX A

### A.1

**Table:**

Core questions	Possible prompts
Part I: Management priorities	
For me to get some background to yourself, I wonder if you could start by talking a bit about your diabetic foot condition?	When were you diagnosed with diabetes? When did your first ulcer occur? How did it come about? Who is involved in your care? How long have you had it for?
I would like now to talk specifically about the activities that you currently do from home to manage your diabetic foot condition. Could you describe this for me?	What activities are done daily and what are done on a weekly basis?
Inevitably, some people would be more confident in managing their condition from home while others are less confident. How confident are you in managing your condition at home?	Could you explain this further and provide me with more details? Could you manage as well without the support of your career?
If you did not feel confident or moderately confident, why did you say so?	What were the problems you encountered? What sort of help did you require?
You mentioned that you are confident in managing your diabetic foot condition. Have you ever encountered any problems?	Did this occur before developing an ulcer? What helped you overcome this problem?
What is the most important outcome for you when it comes to caring for your condition at home?	How does it fit with the other priorities that you have? What do you think are some reasons for that?
Part II: Barriers and facilitators to secondary prevention	
I would like now to explore the challenges you faced in the care of your diabetic foot condition at home. What areas (if any) of your diabetic foot management do you find difficult?	Could you describe this for me? What makes this/these difficult? e.g. physical factors/environment/condition‐related factors/other constraints?
In your opinion, what factors have contributed to success in the management of your diabetic foot condition from home?	Could you explain this further? e.g. Physical factors/environment/condition‐related factors/other constraints ?
What things could be done to make it easier for others with a similar condition to engage with their diabetic foot disease care from home?	Could you explain this further?
Part III: Ideas and preferences of a secondary prevention program	
In your view, what would be some activities or things that could enable you to better manage your diabetic foot condition at home, or support you in your day‐to‐day management activities?	Could you provide me with more details/describe this for me? Could you illustrate with some examples?
Thinking about the activities that you have mentioned above, how do you think this could be best delivered?	Could you provide me with more details/describe this for me? Would you have any preferences?
Are there any issues that would make it difficult to put these supports in place?	Consider physical factors/environment/condition‐related factors/other constraints etc.?
Increasingly, health services are exploring the use of technology in delivery of remote diabetic foot programs/consults to patients. What do you think of such a service delivered remotely?	What are the potential issues you might encounter? What would the ideal delivery of such a service look like? What are some benefits and draw backs of such a service?
